# Laparoscopic adrenalectomy for large adrenal tumor guided by a 3D anatomic model. New frontiers in abdominal surgery

**DOI:** 10.1093/jscr/rjad104

**Published:** 2023-03-09

**Authors:** Alessio Giordano, Francesco Moroni, Giacomo di Filippo, Francesca Cammelli, Tommaso Guagni, Davina Perini, Stefano Cantafio

**Affiliations:** General Surgery Unit, Nuovo Ospedale “S. Stefano”, Azienda ASL Toscana Centro, Via Suor Niccolina Infermiera 20/22, 59100 Prato, Italy; General Surgery Unit, Nuovo Ospedale “S. Stefano”, Azienda ASL Toscana Centro, Via Suor Niccolina Infermiera 20/22, 59100 Prato, Italy; General Surgery Unit, Nuovo Ospedale “S. Stefano”, Azienda ASL Toscana Centro, Via Suor Niccolina Infermiera 20/22, 59100 Prato, Italy; General Surgery Unit, Nuovo Ospedale “S. Stefano”, Azienda ASL Toscana Centro, Via Suor Niccolina Infermiera 20/22, 59100 Prato, Italy; General Surgery Unit, Nuovo Ospedale “S. Stefano”, Azienda ASL Toscana Centro, Via Suor Niccolina Infermiera 20/22, 59100 Prato, Italy; General Surgery Unit, Nuovo Ospedale “S. Stefano”, Azienda ASL Toscana Centro, Via Suor Niccolina Infermiera 20/22, 59100 Prato, Italy; General Surgery Unit, Nuovo Ospedale “S. Stefano”, Azienda ASL Toscana Centro, Via Suor Niccolina Infermiera 20/22, 59100 Prato, Italy

**Keywords:** laparoscopic adrenalectomy, 3D model, large adrenal tumor

## Abstract

If until few years ago the surgeon could study a complex surgery only on the basis of two-dimensional images, today can use 3D physical models on a scale of 1 to 1 of an organ. We report the case of a 53 years old woman with Cushing’s syndrome and a giant right adrenal tumor. To better define the relationship between the neoplasm and inferior vena cava, the vascularization of the adrenal gland, any anatomical anomalies and the specific location of the middle adrenal vein, a 3D printed model was created in 1: 1 size based on the preoperative CT. A laparoscopic right adrenalectomy was performed. No intraoperative and postoperative complications were observed with resolution of the adrenal disorder. This case highlights the feasibility and clinical effectiveness of 3D anatomical models for correct preoperative planning, the surgeon’s intraoperative guidance to reduce possible errors and therefore improve the patient’s postoperative outcome.

## INTRODUCTION

With the increasing development in research and technology, 3D models have developed in many fields, including healthcare, thanks to its ability to reproduce complex geometries, such as that typical of solid organs and major blood vessels. Three-dimensional printing for abdominal surgery is usually related to the creation of physical replicas of a patient-specific anatomical model for the purpose of adequate planning of the surgery but its use can also be extended to the production of surgical instruments or implantable devices [1].

Large adrenal tumors (LAT) are tumors with size more than 6 cm [2] and have an incidence ranging from 8.6 to 38.6% of all adrenal tumors [3]. These are rare cancer associated with malignancy in 25% of cases [4, 5].

We report a case of laparoscopic adrenalectomy performed for right LAT where the creation of a 3D anatomical model made from CT images allowed adequate surgical preparation for the operation.

## CASE REPORT

A 53-year-old obese woman (BMI 35 kg/m^2^) came to our hospital for poorly controlled arterial hypertension and glucose intolerance.

**Figure 1:**
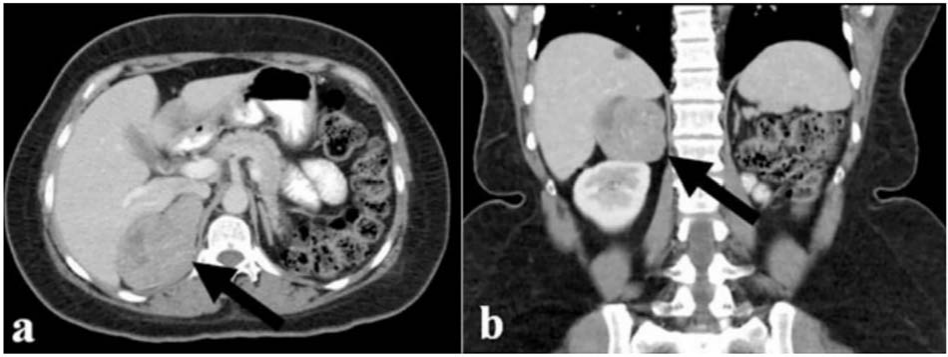
(**a**) and (**b**) The abdominal CT scan showed a large right adrenal tumor.

**Figure 2:**
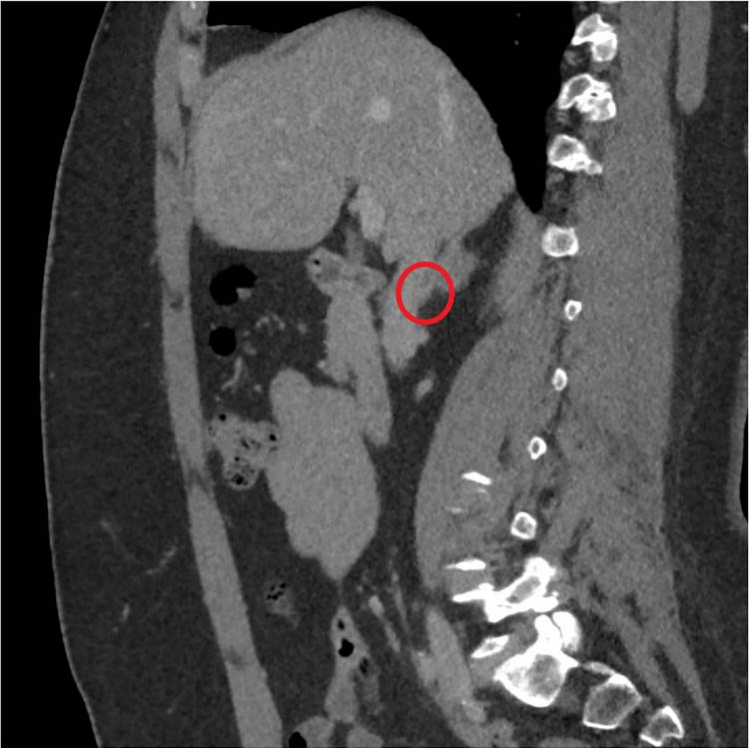
Sagittal 3D reconstruction of CT scan in venous phase. In particular, the images had raised the suspicion of the short course of the adrenal vein in the lower lateral lip of the mass.

The endocrinological study showed a hypersecretion of cortisol with suppressed ACTH (550 mcg/l, ACTH 1.1 ng/ml, urinary free cortisol 255 mcg/24 h, positive overnight Nugent test). The serum concentration of renin and aldosterone and the urinary concentration of metanephrine are instead normal. Therefore, the patient underwent an abdominal CT scan that showed a right adrenal lesion of about 7 cm with an adenomatous appearance (Fig. 1). The saggital 3D reconstruction of CT scan in venous phase had raised the suspicion of short course of the adrenal vein in the lower lateral lip of the mass (Fig. 2). To better define the relationship between the neoplasm and the inferior vena cava, the vascularization of the adrenal gland with any anatomical anomalies and the specific location of the middle adrenal vein, a 3D printed model was created in 1:1 size based on the preoperative CT scan.

**Figure 3:**
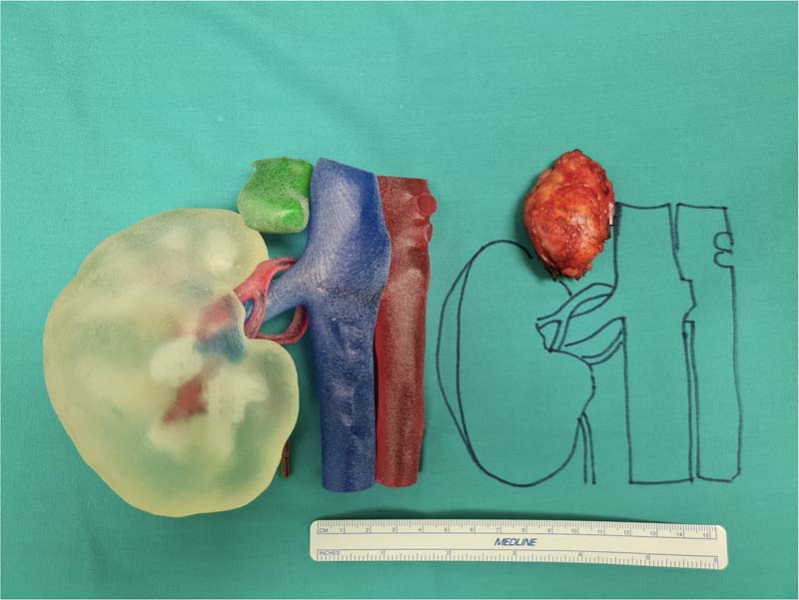
Our 3D anatomic model printed in 1:1 size with the large adrenal tumor removed.

A right laparoscopic adrenalectomy with a lateral transperitoneal approach was performed, after adequate perioperative and postoperative preparation with hydrocortisone to prevent postoperative Addison syndrome. Patient was placed in a lateral decubitus position with the affected side up. This approach allows a wider surgical workspace with an excellent exposure of the upper retroperitoneum, good control of vascular structure if compared with other approaches [5]. We used four trocars in the right subcostal region. As confirmed by the 3D reconstruction, the main adrenal vein had a particularly short course compared with the vena cava, which allowed its adequate dissection without causing any iatrogenic lesions (Fig. 3). The operative time was 65 min and blood loss was 72 ml. One drainage was routinely used in all operations. At the end of the procedure, the large tumor was positioned in endo-bag device and removed with a subcostal mini-laparotomy.

The patient had a regular postoperative course without complications and was discharged on the third postoperative day.

Histological examination confirmed a cortical adenoma.

One month after the surgery, the patient was in good general clinical condition and the cortisol levels were within the limits with improvement in the blood pressure and glycemic control.

The patient underwent an endocrinological follow-up every 6 months and the adrenal hypersecretion disorder was resolved.

## DISCUSSION

Nowadays, modern multi-detector computed tomography and magnetic resonance imaging systems, in combination with the advances in image processing, allow the generation of detailed 3D virtual models through a layer-by-layer process [6]. 3D printing can enable the transition from the virtual world to the real one, providing the tactile feedback of an anatomical model. This technology is nowadays used above all in cardiovascular surgery, vascular neurosurgery, dental surgery, maxillofacial surgery, orthopedics and spinal surgery [9], but in abdominal surgery, we find a fair development in renal and hepatic surgery.

In a recent study, Bianchi et al. [7] evaluate the impact of 3D model for a comprehensive assessment of surgical planning and quality of partial nephrectomy (PN) and in particular the impact of 3D models-based surgical planning on Trifecta achievement, defined as the contemporary absence of positive surgical margin, major complications and ≤ 30% postoperative eGFR reduction. In sum, 195 patients with renal mass scheduled for PN were enrolled in two groups: Study Group (*n* = 100), including patients referred to PN with revision of both 2D computed tomography (CT) imaging and 3D model and control group (*n* = 95), including patients referred to PN with revision of 2D CT imaging. The results were very interesting because a 3D-guided approach to PN increases the adoption of selective clamping and better predicting the achievement of Trifecta. In fact, 73 (80.2%) patients in the Study group and 53 (63.1%) patients in the Control group achieved the Trifecta (*P* = 0.01). The preoperative plan of arterial clamping was recorded as clampless, main artery and selective in 22 (24.2%), 22 (24.2%) and 47 (51.6%) cases in the Study group vs. 31 (36.9%), 46 (54.8%) and 7 (8.3%) cases in the Control group, respectively (*P* < 0.001). At multivariate logistic regressions, the use of 3D model was found to be independent predictor of both selective or super-selective clamping and Trifecta’s achievement.

3D models have been also efficacious in pediatric surgery as a surgical simulation. In their study, Souzaky et al. [8] used 3D printed models based on preoperative CT images of adrenal neuroblastoma (described three cases) to understand the patient’s surgical anatomy and to plan the surgical procedures, especially for determining the optimal port layout.

There are very few cases reported in the literature of 3D prints used in adrenal surgery [9] and demonstrate the importance of this method for the treatment of complex adrenectomies or in the planning of partial adrenectomies with an impact on surgical outcomes. In a recent study, Palomba et al. [10] evaluate the intraoperative role of indocyanine green (ICG) fluorescence associated with preoperative three-dimensional reconstruction (3DR) in laparoscopic adrenalectomy in terms of perioperative outcomes. After propensity score matching analysis, a cohort of 36 patients divided into two groups of 18 patients each was enrolled (standard LA and those undergoing preoperative 3D reconstruction and intraoperative ICG fluorescence). The analysis of these data showed how the operative time and intraoperative blood loss were shorter in patients of the 3DR group (*P* = 0.004 and 0.004, respectively), but there was no difference in terms of the length of stay, conversion rate, and intraoperative and postoperative complications between the two groups.

The possibility of being able to make these models directly from preoperative radiological images allows the following: adequate surgical planning, especially in complex surgery, showing anatomical details and anticipating some technical problems and intraoperative complications, thus improving the patient’s outcome and reducing surgical times; anatomical comprehension and intraoperative navigation: the surgeon can use these graspable objects during surgery to enhance his/her orientation; education and training for young surgeons, facilitating the study of the technical times of a surgical intervention; simulation, thanks to the use of deformable materials that enable dissection, suturing and performance of anastomosis, and, therefore, the surgeon can improve their skills by repeating the procedure multiple times; finally, the surgeon can use the 3D model during the preoperative interview with the patient and his family, illustrating in detail the surgical intervention with its risks and benefits [8, 9].

However, we must certainly think about the costs of these models and it will be necessary to develop suitable materials that mimic the tissue characteristics of the various organs due to the difficulty in reproducing tissue features such as elasticity, softness and tension [11]. For these reasons, the 3D models will certainly be used for specific cases. Several authors mention that the complexity of cases could justify the additional cost of surgical guides [9, 12].

The presence of an LAT with the study of the relationships that the tumor has with the adjacent organs and vascular structures or the execution of partial adrenalectomies could be the fields in which the 3D anatomical models could be used.

The adrenal tumor size criteria remain the subject discussed in literature for the main surgical approach. In accordance with recent records [5] we confirm that adrenal tumor size is only a predictive parameter of possible malignancy. In fact, for lesions greater than 6 cm, the risk of malignancy is 25% (10–53%) [13]. At the moment, there is no evidence that suggests that a laparoscopic approach is contraindicated for LAT because the size is only a predictor factor of malignancy [14, 15]. Conversely, the open approach is recommended in patients with evidence at preoperative imaging studies of malignancy. [16].

## CONCLUSION

If until a few years ago the surgeon could study a complex surgery only on the basis of two-dimensional black and white images, today can have 3D physical models on a scale of 1:1 of an organ or specific anatomical structures for the purpose of adequate planning of the surgical intervention. In the context of adrenal surgery, the 3D models could help the surgeon in case of large lesions to define the best surgical strategy and also as a guide in cases of partial adrenalectomy.

## STATEMENT

The authors state that the work described has not been published previously, it is not under consideration for publication elsewhere and its publication is approved by all authors.

## CONFLICT OF INTEREST STATEMENT

The authors certify that there is no actual or potential conflict of interest in relation to this article and they state that there are no financial interests or connections, direct or indirect, or other situations that might raise the question of bias in the work reported or the conclusions, implications or opinions stated—including pertinent commercial or other sources of funding for the individual author(s) or for the associated department(s) or organisation(s), personal relationships or direct academic competition.

## FUNDING

The authors state that no funding has been received for this article.

## AUTHOR CONTRIBUTION

All authors contributed equally to this work.
